# Public knowledge and awareness of obstructive sleep apnea in Saudi Arabia: A population-based study of over 16,000 adults

**DOI:** 10.1371/journal.pone.0337822

**Published:** 2025-12-12

**Authors:** Abdulelah M. Aldhahir, Mohammed M. Alyami, Abdulhakim M. Aljumayh, Atheer M. Al Omayr, Sahar A. Alghamdi, Ahmed H. Alasimi, Rami A. Alyami, Abdullah A. Alqarni, Jaber S. Alqahtani, Abdallah Y. Naser, Hassan Alwafi, Mohammad S. Dairi, Saeed M. Alghamdi, Mansour S. Majrshi, Rayan A. Siraj, Nowaf Y. Alobaidi, Mohammed A. Almeshari, Sulaiman S. Alsaif, Mushabbab A. Alahmari, Yousef D. Alqurashi

**Affiliations:** 1 Respiratory Therapy Program, Department of Nursing, College of Nursing and Health Sciences, Jazan University, Jazan, Saudi Arabia; 2 Health Research Center, Jazan University, Jazan, Saudi Arabia; 3 Respiratory Therapy Department, Batterjee Medical College, Khamis Mushait, Saudi Arabia; 4 National Heart and Lung Institute, Imperial College London, London, United Kingdom; 5 Department of Respiratory Therapy, Faculty of Medical Rehabilitation Sciences, King Abdulaziz University, Jeddah, Saudi Arabia; 6 Respiratory Therapy Unit, King Abdulaziz University Hospital, Jeddah, Saudi Arabia; 7 Department of Respiratory Care, Prince Sultan Military College of Health Sciences, Dammam, Saudi Arabia; 8 Department of Applied Pharmaceutical Sciences and Clinical Pharmacy, Faculty of Pharmacy, Isra University, Amman, Jordan; 9 Department of Pharmacology and Toxicology, College of Medicine, Umm Al-Qura University, Makkah, Saudi Arabia; 10 Department of Medicine, College of Medicine, Umm Al-Qura University, Makkah, Saudi Arabia; 11 Clinical Technology Department, Respiratory Care Program, Faculty of Applied Medical Sciences, Umm Al-Qura University, Makkah, Saudi Arabia; 12 King Abdulaziz University Hospital, King Abdulaziz University, Jeddah, Saudi Arabia; 13 Department of Respiratory Therapy, College of Applied Medical Sciences, King Faisal University, Al- Ahsa, Saudi Arabia; 14 Respiratory Therapy Department, College of Applied Medical Sciences, King Saud Bin Abdulaziz University for Health Sciences, Alahsa, Saudi Arabia; 15 King Abdullah International Medical Research Centre, Alahsa, Saudi Arabia; 16 Rehabilitation Health Sciences Department, College of Applied Medical Sciences, King Saud University, Riyadh, Saudi Arabia; 17 Department of Respiratory Therapy, College of Applied Medical Sciences, University of Bisha, Bisha, Saudi Arabia; 18 Health and Humanities Research Center, University of Bisha, Bisha, Saudi Arabia; 19 Respiratory Care Department, College of Applied Medical Sciences, Imam Abdulrahman Bin Faisal University, Dammam, Saudi Arabia; UTM Skudai: Universiti Teknologi Malaysia, MALAYSIA

## Abstract

**Background:**

Obstructive sleep apnea (OSA) is a common yet underdiagnosed sleep-related breathing disorder with significant health implications. Despite its clinical relevance, data on population-level knowledge of OSA in Saudi Arabia remain limited.

**Methods:**

A nationwide cross-sectional survey was conducted between November 28th, 2023, and October 18th, 2024, to assess the level of public knowledge and awareness about OSA among the general population in Saudi Arabia.

**Results:**

A total of 16,662 participants completed the survey, with a mean age of 31 years. Obesity (15.8%) was the most commonly self-reported health condition. Most respondents rated their sleep quality as good (36.2%) or acceptable (28.9%), while only 7.5% reported consistent physical activity. Overall, only 12.9% of participants demonstrated good knowledge of OSA. Males were more likely to have good knowledge than females (OR: 1.20, 95% CI: 1.07–1.33, p = 0.001). Residents of the Northern region had significantly higher awareness compared to those in the Central region (OR: 1.39, 95% CI: 1.18–1.64, p < 0.001). Lower educational attainment was associated with reduced awareness: diploma holders (OR: 0.58, 95% CI: 0.50–0.68, p < 0.001) and primary/intermediate education (OR: 0.61, 95% CI: 0.47–0.80, p < 0.001). Former smokers were more knowledgeable than current smokers (OR: 2.03, 95% CI: 1.69–2.44, p < 0.001). Participants with obesity had significantly higher odds of good knowledge compared to those with normal BMI (OR: 1.55, 95% CI: 1.27–1.88, p < 0.001).

**Conclusion:**

Public knowledge about OSA in Saudi Arabia is considerably low, with awareness varying significantly by gender, region, education level, smoking status, and BMI. Targeted public health initiatives are essential to enhance understanding, promote early detection, and improve management of OSA across the population.

## 1. Introduction

Obstructive Sleep Apnea (OSA) is the commonest respiratory sleep disorder, which is characterized by repetitive episodes of upper airway obstruction during sleep, leading to intermittent hypoxia, sleep fragmentation, and daytime dysfunction [1]. Globally, OSA affects approximately almost 1 billion of adults, with rate exceeding 50% in some countries [2]. In Saudi Arabia, epidemiological data indicate that clinically diagnosed OSA affects around 9% of the adult population [3]. This growing prevalence correlates with the rising obesity epidemic in Saudi Arabia, where obesity rates have escalated to 35% among adults [4]. A community-based study demonstrated that lifestyle factors, such as poor dietary habits and sedentary behavior, exacerbate weight gain, contributing to the increasing incidence of OSA [5].

Untreated OSA is significantly associated with a range of comorbidities, including cardiovascular diseases, hypertension, type 2 diabetes mellitus, ultimately leading to increased mortality risk [6]. Additionally, cognitive dysfunction and daytime sleepiness could further complicate the clinical condition, resulting in impaired concentration, memory deficits, and increased risks of vehicular accidents [7,8]. The chronic nature of OSA can also exacerbate psychological distress, contributing to depressive and anxiety symptoms, which profoundly affect the overall quality of life of affected individuals [9]. Furthermore, untreated OSA has also been implicated in increased healthcare utilization, reflecting a higher burden on healthcare systems [10,11].

Despite the alarming prevalence of OSA and its associated health complications, levels of awareness and understanding of the disorder remain disproportionately low among the general population in Saudi Arabia [12,13]. Observational studies reveal a considerable gap in public knowledge regarding OSA’s symptoms, risk factors, and potential health consequences, contributing to substantial underdiagnosis and barriers to effective treatment access [14,15]. Additionally, a study targeting healthcare professionals indicated insufficient training and awareness levels regarding OSA, resulting in a significant knowledge deficit that may obstruct timely diagnosis, referral, and management of the condition [16,17]. Consequently, there is an urgent need for early detection and comprehensive screening methods targeting high-risk populations, supported by public health initiatives aimed at enhancing awareness and understanding of OSA among the general population and healthcare practitioners [18,19]. Considering these pressing concerns, this population-based study aimed to assess the level of public knowledge and awareness about OSA across diverse demographics within Saudi Arabia.

## 2. Materials and methods

### 2.1 Study design and study population

A cross-sectional study was conducted to assess public knowledge and awareness of OSA among the general population in Saudi Arabia between November 28^th^, 2023, and October 18^th^, 2024. Participants were recruited from all regions of the Kingdom, with eligibility limited to Saudi adults (aged ≥18 years) currently residing in Saudi Arabia; non-Saudi residents and minors were excluded.

### 2.2 Study instrument and Sampling strategy

A structured questionnaire was designed by experts in sleep and respiratory medicine to assess the general public’s knowledge and awareness of OSA in Saudi Arabia. The questionnaire, developed in English, consisted of 23 questions divided into two sections. Section I focus on demographic information, such as gender, age, geographical location, education level, smoking status, height, weight, and occupation within the healthcare sector. Body mass index (BMI) was calculated from self-reported height and weight, consistent with previous studies that have validated the accuracy of self-reported anthropometric data for estimating BMI [20,21]. Section II aimed to evaluate awareness of OSA, its risk factors, symptoms, health consequences, and available treatment options. The survey included multiple-choice and Likert-scale questions, allowing participants to select one or more relevant responses where applicable. A first pilot study for the original version was conducted among 15 individuals from both the general population and healthcare providers to assess the content validity of the instrument, with necessary modifications made based on the feedback.

As the questionnaire was originally developed in English, a “forward-backward translation” method was utilized following the World Health Organization [22]. A professional translator with expertise in medical terminology has translated the questionnaire into Arabic. Then, a second professional translator translated the Arabic version back into English. Both versions were compared to identify any differences or inconsistencies. A second pilot study was conducted among 10 individuals from both the general population and healthcare providers to assess the content validity of the instrument, with necessary modifications made based on the feedback [23]. The final version of the questionnaire was distributed in public places and via social media platforms, ensuring broad reach and accessibility across various demographic groups in Saudi Arabia.

### 2.3 Power calculation

With a 99% confidence interval, a 1% margin of error, and an estimated adult population of approximately 35.3 million in Saudi Arabia for 2024, with a 50% response distribution, the minimum required sample size was calculated to be 16,580 respondents [24].

### 2.4 Ethical consideration

Ethical approval for this study was granted by the Research Ethics Committee at King Abdulaziz University on 20 November 2023, before the study commenced (Reference No. FMRS-EC2024–017). The study adhered to the ethical principles outlined in the Declaration of Helsinki. Participation was entirely voluntary, and written informed consent was obtained from all participants before they proceeded with the survey. The study objectives, potential benefits, and the right to withdraw at any time were clearly communicated to ensure informed decision-making. To protect participant confidentiality, all responses were anonymized, and no personally identifiable information was collected. Data were securely stored and accessed only by authorized researchers in compliance with ethical guidelines.

### 2.5 Statistical analysis

Data were analyzed using Stata 17 software. Demographic characteristics were summarized using frequencies and percentages for categorical variables and means with standard deviations (SD) for continuous variables. Knowledge of OSA was assessed using a 10-item survey, where correct answers were scored as 1 and incorrect answers as 0. The total score for each participant was calculated by summing the individual responses. A cutoff score of ≥60% (equivalent to ≥6 correct answers) was used to define “good knowledge,” while scores below 60% indicated “poor knowledge.” This threshold was adopted based on similar frameworks utilized in previous studies evaluating awareness of chronic diseases [25–27], ensuring consistency with established methodologies. Additionally, variables that showed a p-value <0.20 in the univariate analysis were entered into the multivariate model using the enter method to control for potential confounders. Adjusted odds ratios (AORs) with 95% confidence intervals were calculated to identify independent predictors of the OSA awareness. Missing data were minimal (<1%) and were excluded through complete-case analysis.

## 3. Results

### Demographic Characteristics

The study included 16,662 participants, with a nearly equal gender distribution (50.3% female, 49.7% male). The mean age of respondents was 31 years (SD = 10), reflecting a broad age range. Geographically, participants were predominantly from the central region (43.8%), followed by the western (19.7%), eastern (17.5%), southern (10.8%), and northern (8.2%) regions. Educationally, 53.8% held bachelor’s degrees, while 22.2% completed high school, 16.8% held diploma degree, and 4.3% reported primary/intermediate-level education. Body mass index (BMI) was calculated from self-reported height and weight and categorized using standard cutoffs. Based on BMI classification, 7.5% were underweight, 36.3% normal weight, 29.4% overweight, 25.8% had obesity, and 1.0% had severe obesity. A majority (80.4%) did not work in the healthcare sector, and 70.7% identified as non-smokers, with 20.7% reporting current smoking habits. These demographic factors highlight a diverse population with variable exposure to health-related knowledge and OSA risk profiles (Table 1).

**Table 1:** Demographic data of the respondents (n = 16,662).

Variables		Frequency (%)	Mean (± SD)
**Gender**	Female	8374 (50.3%)	
	Male	8288 (49.7%)	
**Age**			31.2 (±10.2)
**Geographical location**	Central region	7303 (43.8%)	
	Eastern region	2908 (17.5%)	
	Northern region	1367 (8.2%)	
	Southern region	1807 (10.8%)	
	Western region	3277 (19.7%)	
**Degree**	Primary/Intermediate degree	717 (4.3%)	
	High school degree	3705 (22.2%)	
	diploma degree	2799 (16.8%)	
	Bachelor’s degree	8963 (53.8%)	
	Postgraduate degree	478 (2.9%)	
**BMI**	Underweight	1254 (7.5%)	
	Normal weight	6044 (36.3%)	
	Overweight	4894 (29.4%)	
	Obesity	4305 (25.8%)	
	Severe Obesity	165 (1.0%)	
**Working in healthcare sector**	No	13393 (80.4%)	
	Yes	3269 (19.6%)	
**Smoking status**	current smoker	3452 (20.7%)	
	Ex smoker	1434 (8.6%)	
	Nonsmoker	11776 (70.7%)	

**-** BMI categories are **derived from calculated BMI** using self-reported height/weight; values are mutually exclusive and sum to ~100%.

### OSA-Associated Comorbidities

Among respondents, separately, 15.8% self-identified obesity as a comorbidity on the checklist (Table 2); this self-report may differ from BMI-derived status. followed by 10.3% experiencing snoring, a hallmark symptom of OSA. Other conditions, such as hypertension (12.2%) and diabetes mellitus (6.6%), were less commonly reported, while chronic heart or respiratory diseases were rare (3.0% each). Notably, 59.4% of participants denied having any of the listed conditions, suggesting a potential under-recognition or under-reporting of OSA-related comorbidities. The high prevalence of obesity, a key OSA risk factor, contrasts with the low rates of diagnosed OSA (20.0% personal/familial diagnosis), highlighting a gap between risk factor presence and clinical identification (Table 2).

**Table 2:** Characteristics of the respondents.

Variables		Frequency (%)
**Comorbidities and symptoms**	Obesity	2628 (15.8%)
	Snoring	1711 (10.3%)
	Chronic Heart Diseases	505 (3.0%)
	Chronic Respiratory Diseases	496 (3.0%)
	Hypertension	2027 (12.2%)
	DM	1097 (6.6%)
	No/ I don’t know	9899 (59.4%)
**Quality of sleep**	Very bad	356 (2.1%)
	Bad	1568 (9.4%)
	Acceptable	4821 (28.9%)
	Good	6025 (36.2%)
	Excellent	3892 (23.4%)
**Exercise frequency**	Never	1615 (9.7%)
	Rarely	4283 (25.7%)
	Sometimes	6356 (38.1%)
	Often	3158 (19.0%)
	Always	1250 (7.5%)

- Comorbidities/symptoms are **self-reported** checklist items**; multiple responses allowed;** percentages need not sum to 100%. ‘Obesity’ here reflects self-identification, **not** BMI-derived classification.”

### Lifestyle Factors and Self-Reported Sleep Quality

A majority of respondents rated their sleep quality as “good” (36.2%) or “acceptable” (28.9%), while 11.5% reported poor sleep (“bad” or “very bad”). Physical activity levels provided additional context: only 7.5% of participants reported always exercising, 19.0% exercised often, 38.1% sometimes, 25.7% rarely, and 9.7% never engaged in exercise. Limited physical activity, a modifiable lifestyle factor linked to sleep hygiene, may contribute to suboptimal sleep patterns even among those who subjectively rated their sleep positively. Notably, despite 59.0% associating OSA with increased risk of accidents, only 16.4% of individuals with poor sleep quality sought medical consultation (Table 2).

### Awareness of Obstructive Sleep Apnea (OSA)

The level of OSA knowledge was classified using a 10-item survey, with a cutoff of **≥**60% correct responses (≥6/10 questions) defining “good knowledge,” consistent with methodologies applied in prior health literacy studies [26–28]. Using this threshold, **2,152/16,662 (12.9%)** participants were classified as having **good knowledge**, while **14,510/16,662 (87.1%)** had **poor knowledge**. A multivariable logistic regression model, adjusted for demographic, geographic, educational, and health-related factors, revealed significant disparities in OSA knowledge (Table 3).

**Table 3:** Factors associated with OSA awareness (n = 16,662).

Factor	Unadjusted OR (95% CI)	p-value	Adjusted OR (95% CI)	p-value
Gender				
Male (vs. Female)	1.25 (1.14-1.37)	<0.001	1.20 (1.07-1.33)	0.001
Geographical Region				
Northern (vs. Central)	1.80 (1.56-2.10)	<0.001	1.39 (1.18-1.64)	<0.001
Eastern (vs. Central)	1.28 (1.13-1.45)	<0.001	1.15 (1.01-1.31)	0.039
Western (vs. Central)	0.94 (0.83-1.07)	0.038	0.88 (0.77-1.00)	0.059
Education				
Diploma (vs. Bachelor’s)	0.51 (0.44-0.59)	<0.001	0.58 (0.50-0.68)	<0.001
Primary/Intermediate (vs. Bachelor’s)	0.59 (0.46-0.77)	<0.001	0.61 (0.47-0.80)	<0.001
Smoking Status				
Ex-smoker (vs. Current)	1.36 (1.15-1.61)	<0.001	2.03 (1.69-2.44)	<0.001
Non-smoker (vs. Current)	0.93 (0.83-1.04)	0.204	1.34 (1.17-1.53)	<0.001
BMI Category				
Underweight (vs. normal)	0.75 (0.62-0.89)	0.002	0.74 (0.60-0.89)	0.002
Overweight (vs. normal)	0.91 (0.75-1.10)	0.031	0.87 (0.71-1.06)	0.017
Obesity (vs. normal)	1.56 (1.30-1.87)	<0.001	1.55 (1.27-1.88)	<0.001

### Factors associated with OSA awareness

Males had 20% higher odds of good OSA knowledge compared to females (OR: 1.20, 95% CI: 1.07–1.33, *p* = 0.001). Geographic disparities were pronounced: residents of the Northern region exhibited 39% greater odds of awareness than those in the Central region (OR: 1.39, 95% CI: 1.18–1.64, *p* < 0.001), while participants in the Eastern region showed 15% higher odds (OR: 1.15, 95% CI: 1.01–1.31, *p* = 0.039). Conversely, the Western region trended toward reduced awareness (OR: 0.88, 95% CI: 0.77–1.00, *p* = 0.059). Lower educational attainment was strongly associated with poorer knowledge: diploma holders (OR: 0.58, 95% CI: 0.50–0.68, *p* < 0.001) and those with primary/intermediate education (OR: 0.61, 95% CI: 0.47–0.80, *p* < 0.001) had significantly lower odds compared to bachelor’s degree holders. (Table 3).

Health behaviors and BMI categories further differentiated awareness. Former smokers had twice the odds of good OSA knowledge compared to current smokers (OR: 2.03, 95% CI: 1.69–2.44, *p* < 0.001), while non-smokers exhibited 34% higher odds (OR: 1.34, 95% CI: 1.17–1.53, *p* < 0.001). Participants with obesity had higher odds of reporting good OSA awareness compared to those in the normal BMI range (OR: 1.55, 95% CI: 1.27–1.88, *p* < 0.001). This finding reflects an association but does not establish whether obesity itself influences awareness or whether related health experiences contributed to greater knowledge. Age (OR: 1.00, 95% CI: 0.999–1.001, *p* = 0.910) and employment in healthcare (OR: 0.99, 95% CI: 0.88–1.11, *p* = 0.837) were not associated with OSA knowledge. (Table 3).

## 4. Discussion

To our knowledge, this is the largest population-based study to assess public knowledge and awareness of OSA in Saudi Arabia. The primary finding of this national survey, which included over 16,000 adult participants with equal gender representation and a broad age range, was that more than 85% of respondents had a concerning poor knowledge of OSA, highlighting a significant gap in public awareness. Female participants and individuals with lower educational attainment were less likely to have good knowledge of OSA, compared to males and those with higher education levels. In contrast, residents of the northern regions, former smokers, and individuals with obesity were more likely to demonstrate higher levels of knowledge about OSA than those residing in other regions, current smokers, and individuals with normal weight.

Despite the increasing prevalence rates, knowledge and awareness of OSA remain concerningly low among the general public [12,13]. The findings of this large national survey highlight the limited and poor knowledge and awareness of OSA in Saudi Arabia. These results are consistent with previous data from a cross-sectional survey of the Saudi general population (n = 1,000 participants), which reported poor knowledge across all assessed domains, including symptoms, risk factors, complications, and treatment methods knowledge [28]. Similarly, a regional survey conducted in Jazan region (n = 523 participants) found that the majority of the study population had limited knowledge of OSA and were unaware of its associated health complications [29]. Moreover, our analysis showed that males had higher odds of possessing good knowledge about OSA compared to females. This finding is consistent with a previous study that similarly reported greater OSA awareness among men [29]. One plausible explanation is that OSA is more commonly diagnosed in males, which may increase their exposure to information through clinical encounters, health education, or social discussions, thereby enhancing their overall awareness and understanding of the condition [30,31].

In examining regional differences in OSA awareness across Saudi Arabia, our study revealed that residents of the Northern region demonstrated greater awareness compared to other regions. This finding contrasts with earlier evidence from the same region; for example, a recent study in Arar, Northern Saudi Arabia, reported that most of the general population had low levels of knowledge about OSA [14]. This suggests that although awareness appeared higher in our sample, gaps in knowledge within the region likely persist. Comparisons with other regions further underscore these disparities. Studies from Jeddah and Jazan have consistently shown particularly low levels of public awareness, with many individuals unable to identify the basic features of OSA or its potential consequences [13,32]. Conversely, a large population-based study found higher awareness in the Central region, indicating that geographic differences in awareness may be influenced by the reach of health education initiatives, access to specialized care, and community engagement with sleep health issues [33]. Collectively, these regional differences highlight the importance of implementing coordinated, nationwide awareness strategies that are adapted to local contexts in order to reduce disparities and improve overall understanding of OSA across the Saudi population.

Educational attainment and health literacy emerged as strong predictors of OSA awareness in this study. Participants with bachelor’s or postgraduate degrees demonstrated significantly greater knowledge compared to those with lower educational levels. This aligns with findings from other regional studies in Saudi Arabia. For instance, Alshehri et al. reported that individuals in the Asir region with higher education were significantly more aware of OSA symptoms and risks [15]. Similarly, Fageeh et al. found that knowledge about OSA was substantially higher among educated participants in the Western region [12]. These findings reinforce the importance of integrating OSA awareness into broader public health and educational initiatives.

Obesity is a well-established and significant risk factor for OSA, with evidence suggesting that approximately 60–90% of adults diagnosed with OSA are overweight [34,35]. Research consistently demonstrates a positive correlation between body mass index (BMI) and both the likelihood and severity of OSA, with higher BMI values associated with increased diagnostic rates and more severe clinical presentations [2,36]. Individuals with obesity are more likely to experience pronounced OSA symptoms, prompting greater interaction with healthcare services and, consequently, increased exposure to education and awareness about the condition [37]. These findings are consistent with our study, which observed that participants with obesity exhibited significantly higher levels of OSA awareness compared to those with normal weight. Moreover, obesity often coexists with comorbidities such as cardiovascular disease and metabolic syndrome, both of which necessitate closer medical attention and may further reinforce awareness of OSA [38,39]. The strong association between obesity and OSA likely enhances the perceived personal relevance of the disorder among individuals with obesity, thereby fostering greater knowledge and understanding of its implications.

Regarding smoking status, our findings indicated that former smokers exhibited a higher level of awareness and understanding of OSA compared to current smokers. This increased awareness may be attributed to the health education received during smoking cessation efforts, which often highlight the risks associated with a range of conditions, including OSA [40–42]. These results are consistent with a recent meta-analysis suggesting that heavy smokers are significantly more susceptible to severe forms of OSA, a risk often recognized only after smoking cessation [43]. The association between smoking and OSA severity further suggests that current smokers may lack a comprehensive understanding of how their smoking behavior negatively impacts respiratory health [44]. In contrast, former smokers, having confronted the long-term consequences of their habits, may be more motivated to seek knowledge about health risks, including those related to OSA [45].

### Strength and limitations

This study represents the largest population-based survey to date assessing public knowledge and awareness of OSA in Saudi Arabia, with over 16,000 participants from all regions of the Kingdom. The large and geographically diverse sample enhances the representativeness and applicability of the findings. The study also offers novel, context-specific insights that can inform national public health strategies on OSA awareness. However, certain limitations should be acknowledged. The cross-sectional design limits causal inference, as data were collected at a single time point. The use of self-reported measures may have introduced information bias, particularly in variables such as height, weight, and health behaviors, which could affect measurement accuracy despite evidence supporting the reliability of self-reported data in previous studies. Additionally, online recruitment may have led to selection bias, potentially underrepresenting individuals with limited internet access and thereby affecting the generalizability of the results. Finally, the use of a non-validated questionnaire to assess OSA knowledge, although appropriate for exploratory large-scale research, underscores the need for future studies to employ validated instruments to ensure reliability and enable cross-study comparisons.

## Conclusions and future directions

This large national survey revealed that more than 85% of the Saudi population has poor knowledge of OSA, despite the high prevalence of OSA-related risk factors. Awareness was particularly low among women and individuals with lower educational attainment, while higher knowledge levels were observed in residents of the Northern region, former smokers, and those with obesity. These findings underscore the urgent need for targeted nationwide educational initiatives, integration of OSA awareness and screening into primary care and public health programs, and further research to evaluate the effectiveness of such interventions. Improving awareness is essential to reducing the burden of undiagnosed OSA and mitigating its associated health and safety risks.
